# First Whole-Genome Characterization of Avian Nephritis Virus 2 of Broiler Chicken from Pará, Brazil

**DOI:** 10.1128/genomeA.00510-18

**Published:** 2018-06-21

**Authors:** Ceyla Maria Oeiras Castro, Elaine Hellen Nunes Chagas, Delana Andreza Melo Bezerra, Sandro Patroca da Silva, Ana Cecília Ribeiro Cruz, Edivaldo Costa Sousa Júnior, René Ribeiro Silva, Hugo Reis Resque, Yvone Benchimol Gabbay, Joana D’Arc Pereira Mascarenhas

**Affiliations:** a Section of Virology, Evandro Chagas Institute, Ministry of Health, Ananindeua, Brazil; b Section of Arbovirology and Hemorrhagic Fevers, Evandro Chagas Institute, Ministry of Health, Ananindeua, Brazil; c Institute of Biological Sciences, Federal University of Pará, Belém, Brazil

## Abstract

Our results show the first full-genome characterization of avian nephritis virus 2 recovered from stools of broiler chickens at a commercial farm located in Benevides, Pará, Brazil. Nucleotide analyses of whole-genome sequences showed the isolate to be a strain of Avastrovirus 2 in the family Astroviridae.

## GENOME ANNOUNCEMENT

Avian nephritis virus (ANV) is classified in the family Astroviridae and was isolated from a normal broiler chick in 1976. Since then, a number of studies have shown the high prevalence and worldwide distribution of ANVs in commercial chicken flocks (1–3). ANVs cause subclinical to severe infections that result in the retardation of a young chicken’s growth due to interstitial nephritis, which may lead to an increase in mortality (1–3). ANVs have also been detected in turkeys and in domestic and wild pigeons (2, 3). Data on the genetic characteristics of ANVs in Brazil are still scarce.

ANVs have a morphology and genomic organization similar to those of astroviruses. ANVs are small nonenveloped viruses with an icosahedral capsid of approximately 30 nm in diameter that protects the genome of single-stranded RNAs larger than 6.9 kb. The genome is divided into 3 open reading frames (ORFs), ORF1a, ORF1b, and ORF2, and is flanked by untranslated regions (2, 3).

In this study, a pooled fecal sample was collected in July 2009 from 41-day-old broiler chickens of the genus Gallus from a commercial farm located in the municipality of Benevides, Pará, Brazil.

DNA sequencing was performed using a read synthesis system. The cDNA library was prepared and sequenced on an Illumina MiniSeq platform using the methodology described in the Nextera XT DNA library preparation kit (4). The sequencing generated 2,761,286 reads, and the genome was assembled using a hybrid methodology of *de novo* assembly and reference mapping with the IDBA-UD algorithm (5) and Geneious version 8.1.9 (6) programs, respectively. By the *de novo* assembly methodology, 9,737 contigs were generated, 21 of which were related to the family Astroviridae, and were subsequently characterized as ANV-2. Currently, there are no ANV-2 strains with complete genomes in NCBI/GenBank; thus, the ANV-1 strain genome (GenBank accession no. HM029238) was used for mapping, which resulted in a single unitig formed by 985 reads. The average coverage was 21.4×, and the GC content was 45.5%. Nucleotide analysis revealed 85.9% identity with the strain used for reference mapping. Comparative analyzes with ANV-1 strains showed a nucleotide identity of 88.5% and amino acid identity of 96.5% in ORF1b. In ORF2, we found a nucleotide identity of 56.8% and an amino acid identity of 52.6%. With regard to ANV-2 strains, we found 57.6% nucleotide identity and 56.4% amino acid identity in ORF2. Our results show the first complete genome characterization of the ANV-2 strain AVE52/ANV2 belonging to the family Astroviridae.

The identification of ANV in broiler chickens corroborates the fact that ANV circulation is common in chickens. These findings highlight the potential for viral metagenomic analysis in searching for new pathogens, emphasizing the importance of viral monitoring in broiler chickens. Of note, these viral agents are associated with significant economic losses in poultry farming. Furthermore, genomic data are important for future studies, which may contribute to the development of vaccines and other control strategies.

### Accession number(s).

The whole-genome sequence reported here has been deposited in GenBank under the accession no. MH028405.
